# Epigenetic reprogramming in small cell lung cancer

**DOI:** 10.20892/j.issn.2095-3941.2022.0343

**Published:** 2022-08-30

**Authors:** Jingyao Chen, Xiangyu Pan, Feifei Na, Xuelan Chen, Chong Chen

**Affiliations:** 1 State Key Laboratory of Biotherapy and Cancer Center, West China Hospital, Sichuan University, Chengdu 610044, China; 2 Department of Thoracic Oncology, West China Hospital, Sichuan University, Chengdu 610044, China

Small cell lung cancer (SCLC), a highly lethal lung cancer sub-type with distinct neuroendocrine-like features, accounts for 10%–15% of all lung cancers. The overall 5-year survival rate remains less than 10%. SCLC is characterized by early metastasis, thus minimizing the potential patient benefits of surgery. In recent decades, the first-line treatment for SCLC has remained chemotherapy combining etoposide and cisplatin (E/P). Despite high rates of response to E/P treatment, SCLC eventually relapses and is almost universally resistant to treatment at recurrence, thus making SCLC a recalcitrant malignancy. Moreover, the limited knowledge regarding the molecular mechanisms underlying SCLC metastasis and resistance greatly hinders improvements in overall SCLC survival. To better understand the molecular mechanisms of SCLC and discover potential therapeutic targets, extensive efforts have continued for decades. Recently, several studies have implicated epigenetic modifications, including histone modifications, DNA methylation, and chromatin accessibility, in SCLC. NFIB has been documented to promote SCLC metastasis through a widespread increase in chromatin accessibility^1^. Of particular note, one recent study has indicated that KMT2C deficiency promotes SCLC metastasis through DNMT3A-mediated epigenetic reprogramming involving both histone and DNA hypomethylation^2^. These studies have revealed that epigenetic reprogramming plays an important role in SCLC. In this review, we summarize and discuss the advances in basic and translational research in SCLC, which have revealed the functional and targetable roles of epigenetic modifications during tumorigenesis, metastasis, and chemoresistance.

## Epigenetic reprogramming in SCLC initiation and progression

**Figure 1:**
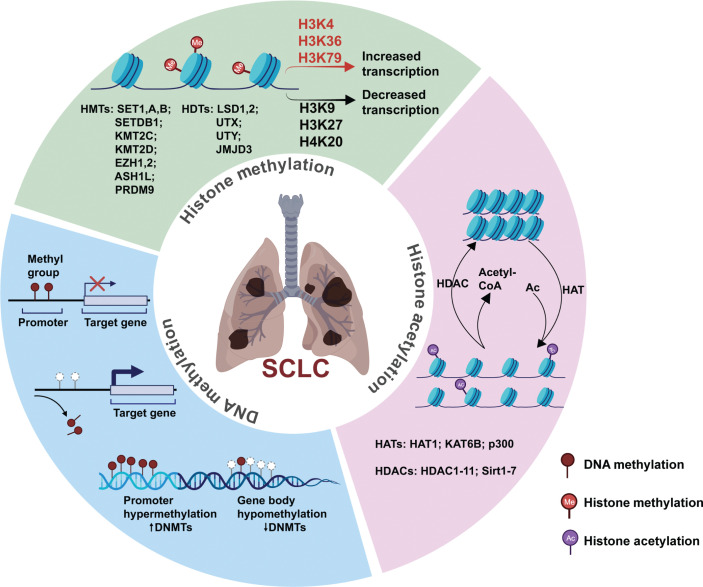
Epigenetic reprogramming in SCLC. Alterations in epigenetic modifications appear at various stages of tumorigenesis in SCLC, including initiation, metastasis, and drug resistance. Epigenetic regulations play an important role in SCLC, mainly through DNA methylation, histone acetylation, histone methylation and chromatin remodeling. Epigenetic regulation has reversible effects on gene silencing and activation *via* translational and post-translational modifications. DNMTs, DNA methyltransferases; HMTs, histone methyltransferases; HDTs, histone demethyltransferases; HATs, histone acetylases; HDACs, histone deacetylases.

Modifications to DNA and histones are critical components of epigenetic regulation in the initiation and progression of SCLC (**Figure 1**).

### DNA methylation

DNA methylation, mainly including 5-methylcytosine and 5-hydroxymethylcytosine, is involved in gene transcription. DNA methyltransferases establish and maintain methylation patterns. Aberrant DNA methylation can result in multiple diseases, particularly inflammatory diseases and cancers. Accumulating evidence suggests that the inappropriate DNA methylation is a signature of SCLC. Compared with other lung cancers, SCLC is notable for its dense clustering of high-level methylation in discrete promoter CpG islands^3^. Kalari et al.^4^ have conducted genome-scale analysis of DNA methylation changes in SCLC and discovered hundreds of tumor-specifically methylated genes. These frequently methylated genes, including *NKX2-1* and *SOX1*, have the potential to be used as biomarkers for SCLC. In addition, DNA hypomethylation has been identified as a biomarker predicting the response rate of SCLC undergoing treatment with LSD1 inhibitors^5^.

### Histone modifications

Histone modifications, mainly acetylation and methylation, modulate transcription and DNA repair, and also maintain chromatin stability. Recent genomic studies have identified many genetic alterations in histone modification regulators in SCLC. The histone methyltransferase genes *KMT2C* and *KMT2D*, and the histone acetyltransferase gene *KAT6B*, are frequently mutated in SCLC. KAT6B, a histone 3 Lys23 acetyltransferase, has been demonstrated as a tumor suppressor in SCLC^6^. However, the lack of clinical SCLC samples and animal models recapitulating features in patients still limits understanding of the molecular mechanisms of histone modification regulatory genes in SCLC initiation and progression.

### Combination of DNA and histone modifications

Metastasis, a hallmark of cancer, is a lethal behavior during cancer progression. Previous studies have revealed that epigenetic alterations drive metastasis in several human cancers, including hepatocellular carcinoma and pancreatic cancer. SCLC is notorious for its early and frequent metastases: more than 70% of individuals with SCLC have metastases at diagnosis^7^. Few genetic mutations driving SCLC metastasis have been reported and validated. Hence, SCLC tumor cells gain epigenetic “drivers” during metastatic progress. Previous studies have used SCLC cell lines to investigate SCLC metastasis-associated epigenetic mechanisms. For instance, NFIB, a transcription factor (TF), promotes metastasis through a widespread increase in chromatin accessibility^1^. NFIB promotes the expression of metastasis-promoting neuronal genes. However, studies have shown that some SCLCs arising from mature neuroendocrine cells become metastatic in the absence of *NFIB*-driven chromatin alterations^8^. Thus, the mechanisms of SCLC metastasis require further study for identification and validation.

A recent study published in *Nature Cancer* by Na et al.^2^ has presented an epigenetic mechanism underlying SCLC metastasis. Using genome-edited lung organoids, the authors developed a primary, orthotopic, and driver-defined SCLC mouse model that faithfully recapitulates the metastatic features observed in patient with SCLC. This model provides a faster orthotopic and primary *in vivo* system to study the mechanisms of SCLC metastasis. In SCLC, KMT2C deficiency promotes multiple-organ metastases in mice. *KMT2C*, encoding a histone 3 lysine 4 (H3K4) mono- and dimethyltransferase, is an epigenetic regulatory gene. In patients, *KMT2C* is frequently mutated in extensive-stage SCLC, particularly in metastatic samples. This study has revealed that KMT2C deficiency initiates epigenetic reprogramming of both histone and DNA methylation underlying SCLC metastasis, thereby suggesting a potentially targetable susceptibility of this malignancy.

KMT2C deficiency is associated with the downregulation of gene expression. *KMT2C* has been revealed to be a tumor suppressor gene, as well as a crucial epigenetic regulator of metastasis in SCLC. Intriguingly, KMT2C directly binds multiple regions of the *DNMT3A* locus and upregulates the expression of *DNMT3A* through increasing histone methylation and chromatin accessibility. *DNMT3A* encodes a DNA methyltransferase, and its loss can lead to global DNA hypomethylation, which is correlated with the activation of gene expression. RNA-seq has revealed that KMT2C deficiency not only represses the expression of metastasis-associated tumor suppressor genes but also up-regulates metastasis-promoting genes. The direct link between KMT2C and DNMT3A clarifies the paradox of how the loss of KMT2C increases the expression of metastasis-associated genes in SCLC, such as *MEIS2* and several *HOXB* genes. Functionally, *Meis2* loss significantly inhibits the metastatic ability of SCLC cells *in vitro* and *in vivo*. *MEIS2/HOXB* genes have been identified as important mediators of KMT2C–DNMT3A loss in driving SCLC metastasis. This study has provided an example of how an epigenetic regulator induces tumorigenesis through concerted epigenetic reprogramming of both histone and DNA hypomethylation.

During SCLC metastasis, KMT2C loss initiates a global decrease in chromosome accessibility, which is associated with decreased H3K4 methylation. However, the mechanism of NFIB in SCLC metastasis involves increased widespread chromatin accessibility. These 2 shifting patterns of chromatin accessibility might be associated with the high heterogeneity of SCLC. The cell type of tumor origin influences the trajectory of tumor progression and cancer types, and even leads to intertumoral heterogeneity in SCLC. To date, 4 SCLC subtypes have been well defined, on the basis of the high expression of the key TFs ASCL1, NEUROD1, POU2F3, and YAP1^9^. Therefore, the intertumoral heterogeneity and cancer subtypes in SCLC profoundly influence the epigenetic and genetic changes underlying SCLC metastasis. Further studies, including epigenetic modification and chromatin accessibility assays in mouse models and patient samples, will be required to determine whether different SCLC subtypes exhibit different epigenetic characteristics and vary in their responses to epigenetic therapies.

## Epigenetic reprogramming in the chemoresistance of SCLC

Although SCLC initially responds very well to the first-line treatment, it eventually relapses and becomes almost universally resistant to E/P treatment after recurrence. Cell-intrinsic alterations, including genetic and epigenetic dysregulation, can drive tumor cells’ chemoresistance. We propose that the rapid epigenetic switch in genome structure and expression level of a large portion of genes gives rise to SCLC chemoresistance (**Figure 1**) for the following reasons. (1) Despite many attempts, between chemoresistant tumors and their primary tumors, few mutations and copy number variations have been found. (2) Gene mutations require long-term accumulation and selection, whereas the progression from chemosensitivity to chemoresistance in patients with SCLC spans less than 6 months. These clues suggest that gene mutations and copy number variations might not be the major factors underlying SCLC chemoresistance.

Histone modification plays an important role in acquired chemoresistance in SCLC. A breakthrough in the study of SCLC chemoresistance has indicated that an EZH2-SLFN11 axis regulates chemosensitivity relapse in SCLC^10^. The high expression of *SLFN11*, a factor implicated in DNA damage repair deficiency, confers increased sensitivity to chemotherapy. EZH2 directly regulates the expression of *SLFN11* through increasing H3K27me3, inducing local chromatin condensation, and eventually causing gene silencing. The inclusion of an EZH2 inhibitor with standard cytotoxic therapies has been found to prevent the emergence of chemoresistance and augment chemoresponse in a mouse model. That study has pioneered understanding of the histone modification dynamics in SCLC chemoresistance and laid an important foundation for clinical research.

Recently, alterations in TFs have been implicated in SCLC chemoresistance. TFs can cause gene-specific transcriptional activation or repression through direct interactions with the promoter regions of downstream targeted genes. The MYC family proteins, including MYCN, MYCL, and MYC, are well-known for their function as TFs. They participate in tumor initiation, maintenance, and progression. Upregulation of the MYC family has also been documented in the chemoresistance of SCLC and other cancers^11^. One recent study has indicated that the activation of Yap, the key TF in the Hippo signaling pathway, enhances chemoresistance in SCLC tumor cells^12^. Yap directly regulates *Notch2* and *Rest* expression in SCLC. Additionally, activation of WNT signaling has been observed in SCLC relapse samples^13^. Recent studies have revealed that the expression of many TFs is altered during SCLC chemoresistance. TFs have been demonstrated to be involved in multiple epigenetic regulatory mechanisms, especially in modulating the DNA methylation landscape. Studies have strongly suggested that epigenetic reprogramming of upstream and/or downstream TFs might be the major driver of SCLC chemoresistance.

## Therapeutic targeting of the SCLC epigenome

Epigenetic reprogramming is a novel hallmark of cancer and is involved in multiple cancer behaviors, particularly tumorigenesis and tumor maintenance. To weaken the influence of abnormal epigenetic reprogramming, efforts in drug development have increasingly focused on targeting epigenetic regulators (**Table 1**). Several epigenetic-targeted therapies have already been approved for the treatment of cancers by the U.S. Food and Drug Administration, and some drugs have exhibited viable therapeutic potential for SCLC in preclinical and clinical trials.

**Table 1:** Key studies of epigenetic drugs in SCLC

Compound	Target	Combined agent	Rationale	Cancer type	Status	Reference
Romidepsin	HDAC	NA	Inhibition of histone deacetylation	Relapsed SCLC	Clinical trial (NCT00086827), phase II, completed	^14^
Pracinostat	HDAC	NA	Inhibition of histone deacetylation	Crebbp-deleted SCLC	Mouse *in vivo* treatment	^15^
Panobinostat/LBH589	HDAC	NA	Inhibition of histone deacetylation	SCLC	Clinical trial (NCT01222936), phase II	^16^
GSK2879552	LSD1/KDM1A	NA	Inhibition of H3K4 and H3K9 demethylation	SCLC	Cell line *in vitro* treatment and PDX *in vivo* treatment	^5^
GSK2879552	LSD1/KDM1A	NA	Inhibition of H3K4 and H3K9 demethylation	Relapsed/refractory SCLC	Clinical trial (NCT02034123), phase I, terminated	^17^
Olaparib	PARP	Temozolomide	Inhibitor of poly(ADP-ribose) polymerase	Relapsed SCLC	Clinical trial (NCT 02446704), phase I and phase II, active, not recruiting	^18^
Veliparib	PARP	Temozolomide	Inhibitor of poly(ADP-ribose) polymerase	Relapsed-sensitive or refractory SCLC	Clinical trial (NCT01638546), phase II, completed	^19^
Veliparib	PARP	E/P	Inhibitor of poly(ADP-ribose) polymerase	Extensive-stage SCLC	Clinical trial (NCT01642251), phase II, completed	^20^
EPZ011989	EZH2	E/P	Inhibition of H3K27me3	Both chemosensitive and chemoresistant models of SCLC	PDX *in vivo* treatment	^10^
SAM	NA	NA	Increase in DNA 5-methylcytosine, H3K4me1, and H3K4me2 levels	SCLC	Mouse *in vivo* treatment and patient sample *in vitro* treatment	^2^

PDX, patient-derived xenograft; NA, not available.

Epigenetic drugs target chromatin regulators, thereby modulating DNA and chromatin structure, and influencing transcriptional and post-transcriptional modifications. Histone deacetylase inhibitors (HDACi), which cause large-scale changes in gene expression, have been evaluated for cancer treatment in pre-clinical and clinical studies. A clinical trial of HDACi in chemosensitive recurrent SCLC was performed more than 10 years ago without success^14^. However, recent research has revealed that the inactivation of *CREBBP*, encoding an acetyltransferase, enhances SCLC sensitivity to HDAC inhibition. This study has suggested a new avenue to select patients with *CREBBP*-mutant SCLC for HDACi treatment^15^. Furthermore, lysine demethylase 1 (LSD1, also known as KDM1A) is a histone modifying enzyme responsible for demethylating H3K4. The LSD1-targeted drug GSK2879552 has been found to have efficient antitumor activity in SCLC cell lines and primary samples. Intriguingly, DNA hypomethylation has been used as a predictive biomarker of activity in response to GSK2879552, thus further suggesting that epigenetic reprogramming plays an important role in SCLC^5^. A clinical trial of GSK2879552 in relapsed/refractory SCLC was terminated because of poor disease control rates and high rates of adverse events^17^. Hence, LSD1 requires further investigation as a therapeutic target for SCLC.

Olaparib, an inhibitor of poly(ADP-ribose) polymerase (PARP), combined with temozolomide, has been found to be a promising new therapeutic strategy in relapsed SCLC^18^. The inclusion of an EZH2 inhibitor with E/P strategy has been found to prevent the emergence of acquired resistance and augment chemotherapy response in a model of chemoresistant SCLC^10^. S-Adenosyl methionine (SAM), the substrate of methyltransferases, plays a critical role in the transfer of methyl groups to various biomolecules, including DNA and histones. It has been approved as a clinical drug for liver cirrhosis, osteoarthritis, and other syndromes. Na et al.^2^ have reported that metastatic and KMT2C-deficient SCLC displays both histone and DNA hypomethylation. SAM repairs these epigenetic abnormalities through significantly increasing the levels of DNA 5-methylcytosine and H3K4me1 and H3K4me2 in *KMT2C*-deficient SCLC. Furthermore, SAM treatment has been found to repress SCLC progression and metastasis in mouse-model and patient samples.

At present, few drugs can be used for patients with SCLC who are resistant to the first-line chemotherapy. Fortunately, increasing studies show that targeting epigenetic regulation is an important direction in anti-SCLC therapy. Clinical trials combining epigenetic drugs with checkpoint blockade and/or chemotherapy will be major avenues for future investigations.

## Conclusions

Epigenetic reprogramming is involved in tumorigenesis, metastasis, and acquired chemoresistance of SCLC. Although only several epigenetic regulators and epigenetic drugs have been reported and designed, teams and studies are increasingly identifying the epigenetic mechanisms underlying multiple cancers, particularly SCLC. Epigenetic reprogramming processes, particularly those involved in DNA methylation, histone modification, and chromatin accessibility, cause large-scale changes in gene expression. Meanwhile, genomic alterations in epigenetic regulatory genes also shape the epigenetic landscape. With the advent of new techniques, understanding of the epigenetic mechanisms in SCLC is expected to be advanced and validated in the near future. More novel findings will provide a roadmap for developing therapeutic strategies targeting epigenetic reprogramming, including combinations with standard chemotherapy and/or immunotherapy.
